# Epigenetic differences at the *HTR2A* locus in progressive multiple sclerosis patients

**DOI:** 10.1038/s41598-020-78809-x

**Published:** 2020-12-17

**Authors:** Vicki E. Maltby, Rodney A. Lea, Sean Burnard, Alexandre Xavier, Thao Van Cao, Nicole White, Daniel Kennedy, Kira Groen, Katherine A. Sanders, Rebecca Seeto, Samara Bray, Melissa Gresle, Louise Laverick, Helmut Butzkueven, Rodney J. Scott, Jeannette Lechner-Scott

**Affiliations:** 1 School of Medicine and Public Health, University of Newcastle, Callaghan, NSW 2308 Australia; 2 Centre for Brain and Mental Health, Hunter Medical Research Institute, New Lambton Heights, NSW 2305 Australia; 3 Institute of Health and Biomedical Innovations, Genomics Research Centre, Queensland University of Technology, Kelvin Grove, QLD 4059 Australia; 4 School of Biomedical Sciences and Pharmacy, University of Newcastle, Callaghan, NSW 2308 Australia; 5 Centre for Anatomical and Human Sciences, Hull York Medical School, Hull, UK; 6 School of Environmental and Life Sciences, University of Newcastle, Callaghan, NSW 2308 Australia; 7 Department of Medicine, University of Melbourne, Melbourne, VIC Australia; 8 Royal Melbourne Hospital, Melbourne, VIC Australia; 9 Alfred Hospital, Melbourne, VIC Australia; 10 MS and Neuroimmunology Unit, Central Clinical School, Monash University, Melbourne, VIC Australia; 11 Division of Molecular Genetics, Pathology North, John Hunter Hospital, New Lambton Heights, NSW 2305 Australia; 12 Centre for Cancer Research, Hunter Medical Research Institute, New Lambton Heights, NSW 2305 Australia; 13 Department of Neurology, John Hunter Hospital, New Lambton Heights, NSW 2305 Australia

**Keywords:** Medical research, Multiple sclerosis

## Abstract

The pathology of progressive multiple sclerosis (MS) is poorly understood. We have previously assessed DNA methylation in the CD4^+^ T cells of relapsing–remitting (RR) MS patients compared to healthy controls and identified differentially methylated regions (DMRs) in *HLA-DRB1* and *RNF39*. This study aimed to investigate the DNA methylation profiles of the CD4^+^ T cells of progressive MS patients. DNA methylation was measured in two separate case/control cohorts using the Illumina 450K/EPIC arrays and data was analysed with the Chip Analysis Methylation Pipeline (ChAMP). Single nucleotide polymorphisms (SNPs) were assessed using the Illumina Human OmniExpress24 arrays and analysed using PLINK. Expression was assessed using the Illumina HT12 array and analysed in R using a combination of Limma and Illuminaio. We identified three DMRs at *HTR2A*, *SLC17A9* and *HDAC4* that were consistent across both cohorts. The DMR at *HTR2A* is located within the bounds of a haplotype block; however, the DMR remained significant after accounting for SNPs in the region. No expression changes were detected in any DMRs. *HTR2A* is differentially methylated in progressive MS independent of genotype. This differential methylation is not evident in RRMS, making it a potential biomarker of progressive disease.

## Introduction

Multiple Sclerosis (MS) is an inflammatory and neurodegenerative disease of the central nervous system (CNS). The majority of patients are initially diagnosed with relapsing–remitting MS (RRMS), which is thought to involve migration of self-reactive immune cells into the CNS^1^. These cells initiate an immune response, which results in localized demyelination and corresponding neurological symptoms^2^. Over a period of time, patients may develop progressive MS, which is characterized by compounding neurodegeneration and a decreased ability to repair damage, resulting in increasing disability. This phase can be active with superimposed relapses or non-active without relapses^1,3^. The role of inflammation in progression is less clear and it is presumed that both immunological and neurobiological mechanisms are responsible for the accumulating damage^1^.

The underlying pathogenesis of MS remains unclear but the prevailing hypothesis is that MS develops due to a combination of genetic predisposition and environmental factors. Epigenetics can influence the genome without changing the DNA sequence. Environmental exposures, such as smoking, have been demonstrated to influence epigenetic patterns^4,5^. One such epigenetic change is DNA methylation, which refers to the addition of a methyl group to a CpG dinucleotide. We, and others, have previously assessed the genome-wide DNA methylation profile in the CD4^+^ and CD8^+^ T cells and CD19^+^ B cells of RRMS patients and compared to that of healthy controls^6–12^. In CD4^+^ T cells, we found a striking methylation signal located at the MHC locus with peaks at *HLA-DRB1* and *RNF39* present in RRMS patients compared to healthy controls^8,11^. We did not identify either of these DMRs in either CD8^+^ T cells or CD19^+^ B cells of MS patients^6,7^. A more recent study investigated the interplay between DNA methylation and genetic background in the *DRB1* region and found that MS patients who were carriers of the high risk HLA-DRB1 15*01 allele had significantly lower DNA methylation at *HLA-DRB1* compared to those who were not carriers. This was the case in monocytes, B cells and both CD4^+^ and CD8^+^ T cells. They further demonstrated a function relationship between genetic background, lowered methylation and increased expression in this region. Thus, the results seen in CD4^+^ T cells may have been due to genetic background^13^.

Our understanding of RRMS has improved markedly. This has translated into new treatment options that target T and B lymphocytes that are also effective in reducing disability progression (reviewed in^14^). Unfortunately, our knowledge of factors that drive MS progression and resulting disability is lacking. A better understanding of disease progression is needed to track disease activity and progression accurately for patients. This is important because disease progression and accompanying disability may not be targeted by current therapies.

Identifying epigenetic loci associated with MS, particularly progressive MS, could reveal potentially important biomarkers of disease course as well as modifiable targets for new drug design.

## Methods

### Ethics approval and consent to participate

The Hunter New England Health Research Ethics Committee and the University of Newcastle Ethics Committees approved this study (2019/ETH12346 and H-505-0607 respectively), and methods were carried out in accordance with institutional guidelines on human subject experiments. Written and informed consent was obtained from all patient and control subjects.

### Sample collection

Whole blood was collected from a discovery cohort of 23 female secondary progressive MS patients (hereafter referred to as progressive MS) and 16 age-and gender-matched healthy controls and an independent validation cohort of 11 female progressive MS patients and 12 age- and gender-matched healthy controls (Table 1). All patients were diagnosed with MS according to the 2017 McDonald criteria^15^. Progressive disease stage was evaluated by the Neurology Staff Specialists, who are EDSS certified, at the John Hunter Hospital or Royal Melbourne Hospital. As there is currently no “gold standard” for defining progression, we have used the most recent recommendations for progression set by the MSBase Study Group^16^. To meet these criteria progressive patients must: (i) have a minimum expanded disability status scale (EDSS) score of 4, (ii) have had disability progression by 1 EDSS step for patients with EDSS ≤ 5.5 or 0.5 for patients with EDSS ≥ 6 in the last year, (iii) have progression in the absence of relapse, (iv) have confirmed progression over ≥ 3 months^16^. All patients had not had MS-specific therapy or steroid treatment for a minimum period of 6 months prior to collection.

**Table 1:** Patient characteristics.

	HC discovery cohort	MS discovery cohort	HC replication cohort	MS replication cohort
Methylation arrays (N)	16	23	12	12
SNP arrays (N)	13	22	0	0
Expression arrays (N)	16	22	0	0
Female	16	23	12	12
Age in years (mean ± SD)	58 ± 9.5	57.9 ± 9.5	61.0 ± 8.6	60.2 ± 9.0
EDSS (mean ± SD)	N/A	6.5 ± 1.3	N/A	6.1 ± 1.1
Disease duration in years (mean ± SD)	N/A	24.5 ± 10.3	N/A	26.5 ± 12.5
Progression duration in years (mean ± SD)	N/A	8.8 ± 5.6	N/A	5.9 ± 4.1

*SD* standard deviation, *HC* healthy controls, *EDSS* expanded disability status score, *N/A* not applicable.

### Sample preparation

Peripheral blood mononuclear cells (PBMCs) were isolated from whole blood by density-gradient centrifugation on Lymphoprep (StemCell Technologies, Canada). CD4^+^ T cells were isolated using negative selection magnetic separation kits (StemCell Technologies, Canada) according to the manufacturer’s protocols. Purity was assessed using a FITC-conjugated anti-CD4 antibody (anti-human CD4 antibody, clone OTK4, catalog #60016F1, StemCell Technologies, Canada) on a FACSCanto II flow cytometer (BD Biosciences, USA) and analyzed using the FACSDiva software (BD Biosciences, USA). All samples met a minimum purity threshold of ≥ 90%.

Extraction of genomic DNA was performed using the QiaAMP DNA micro kit (Qiagen, USA). Processing of the discovery cohort DNA for the Infinium HumanMethylation450 arrays including bisulphite conversion, was done at the Centre for Clinical Genomics (University of Queensland). Processing of the validation cohort samples for Infinium MethylationEPIC arrays was done at the Australian Genome Research Facility (AGRF). The AGRF also performed the Illumina HumanOmniExpress-24 arrays, on the samples with sufficient DNA remaining. Exact numbers of individuals used for each assay are listed in Table 1.

RNA was extracted from samples with sufficient PBMCs using the QiaAMP RNA/miRNA kit (Qiagen, Netherlands). Quality control was assessed using the Qubit fluorometer (quantification) and the Agilent bioanalyzer, using the small and nano RNA kits. Processing of the RNA for Illumina Human HT12 arrays (hereafter referred to as HT12 arrays) was performed by the Centre for Clinical Genomics (University of Queensland).

### DNA methylation analysis

Raw methylation data was analysed primarily using the ChAMP package in R, with the support of several auxiliary scripts (version 2.16.2)^17^. For quality control (QC) assessments, raw methylation data were used to produce beta density plots, multiple dimensional scaling (MDS) plots and sex check plots, each containing assigned phenotype labels (Suppl. Files 1a). To adjust for background and dye bias from different arrays (450 K and EPIC), the raw data was normalized using the single-sample Noob method (ssNoob), which was applied using the “preprocessNoob” function in the Minfi package (version 1.32.0). The ssNoob script was designed to perform background correction for Illumina Methylation data generated for different arrays (ie. 450K and EPIC)^18^. Following normalisation, the QC metrics were re-checked to confirm an improvement in the beta distributions (Suppl. Files 1b). The next step involved removal of bad probes. This included probes with a detection P-value > 0.01, beadcount < 3 in at least 5% of samples, NoCG, SNPs, MultiHit and probes on XY chromosomes. For this step the Minfi and WateRmelon packages (version 1.30.0) were required to generate the necessary data derived from the ssNoob analysis to run the “champ.filter” function^19^. Batch effect analysis was then performed for both cohorts combined using the “champ.SVD” function. The harmonised data set was then adjusted for significant batch effects using the “champ.runCombat” function (Suppl. Files 2).

### Differentially methylated positions and regions

The final harmonised beta value matrix was then moved forward to analysis of differentialy methylated positions (DMPs) and differentialy methylated regions (DMRs). We implemented Champ.DMP and Champ.DMR(DMRCate) to identify DMPs and DMRs respectively. The default false discovery rate of 0.05 was used. Given the modest sample size we also chose to apply selection criteria based on both unadjusted statistical significance and effect size. For this, DMPs were selected if a CpG yielded both an unadjusted P-value less than 0.05 and a Δ_meth_ $\ge$ 0.1 (representing 10% differential methylation). For DMRs we focused only on regions which had ≥ 2 DMPs that mapped to the same gene, were separated by < 1500 bp distance, and where adjacent CpGs yielded Δ_meth_ in a consistent direction. This approach has been successfully applied in previous studies to identify DMPs in small samples^6–8,11^ All data was processed and analyzed in Rstudio (version 1.1.456)^20^ and R (version 3.5.2)^21^.

### Single nucleotide polymorphism (SNP) analysis

Genomic DNA was extracted from whole blood and genotyped using the Illumina HumanOmniExpress-24 array. PLINK (v1.9)^22^ was used for quality control procedures and SNP frequency statistics. Samples were excluded for (1) sex inconsistencies or (2) call rate < 95%. Individual SNPs were removed if (1) the genotype call rate was below 98%, (2) the minor-allele frequency was less than 0.05 and (3) there was significant deviation from the Hardy–Weinberg permutation test (P < 1 × 10^–5^). SNPs were extracted at least ± 5 Kb upstream and downstream of HTR2A, from 47,400,677 bp to 47,477,087 bp (hg19). The progressive MS cohort included 22 cases and 13 controls and the RRMS cohort included 24 cases and 17 controls. A total of 48 variants passed QC and filtering. Fisher’s Exact testing was performed to assess SNP frequencies. Haploview (v.4.2) was used to assess haplotypes^23^.

### Methylation quantitative trait loci (QTL) analysis

Genetic changes can mediate the association between DNA methylation and disease via mQTLs. To explore the possibility of this type of association for key DMRs, we compared mean methylation of CpGs among genotype groups for index SNPs using ANOVA. To assess the conjoint effects of CpG methylation and SNP genotype on MS outcome we used binary logistic regression models.

### Transcriptome analysis

RNA was extracted for gene expression analysis and was measured using Illumina Human HT12 v4 arrays. The programme R (v.3.6.3)^21^ was used with the R packages limma^24^ and illuminaio^25^ and following the Kebschull et al.^26^ pipeline to pre-process, quality control and normalise the idat files. After normalisation, the HTR2A probe value for all samples was extracted and compared.

## Results

Following the QC analysis, the resultant methylation datasets were comprised of 407,400 CpGs for the discovery cohort and 743,477 CpGs for the replication cohort. Of these 378,188 CpGs were common to both cohorts. Batch effect analysis conducted using the SVD function in ChaMP indicated significant methylation clustering by “slide” and “cohort” on methylation profiles (P < 1 $\times$ 10^–5^). These factors were diminished to negligible effects after adjustment using the ComBat normalisation function (Suppl. Files 2).

### Differentially methylated positions (DMPs)

We analysed methylation data from the CD4^+^ T cells obtained from our discovery cohort of 23 female progressive MS patients and 16 female healthy controls. ChampDMPs did not reveal any DMPs that were statistically significant at an FDR of 0.05. This is not surprising given the modest sample size used here. For this reason, we also employed a selection algorithm based on an unadjusted P-value < 0.05 and Δ_meth_. These steps resulted in 72 DMPs that mapped to 34 genes and 23 unannotated genomic locations (Table S1). Of these, 74% were hypermethylated and 26% were hypomethylated with majority located within the gene body or intragenic regions.

We separately analysed the validation cohort of 11 progressive female MS patients and 12 healthy female controls and identified 65 DMPs which mapped to 32 genes and 22 unannotated genomic locations (Table S2). Again, there was a tendency towards hypermethylation, with 68% of DMPs being hypermethylated and the majority of DMPs located within the gene body or intragenic region.

To investigate the level of similarity between the two cohorts, we compared the lists of DMPs identified in both MS cohorts. This revealed 12 DMPs common to both cohorts, which spanned three genes (*HTR2A*, *SLC17A9* and *HDAC4*) and four unannotated regions on chromosomes 12, 16, 5, and 3 (Tables S1, S2 asterisk). All DMPs were altered in a consistent direction (five hypomethylated and seven hypermethylated).

### Differentially methylated regions (DMRs)

Results of DMRCate did not reveal any significant DMRs at an FDR of 0.05. We therefore focused only on regions which had ≥ 2 DMPs located within a 1500 bp distance, where adjacent CpGs yielded Δ_meth_ in a consistent direction. In the discovery cohort, this identified 9 DMRs (Table 2). The largest DMR was found at *MDGA1*, which contained four sites located in the gene body within 1546 base pairs. These sites are contained within a CpG island and have a Δ_meth_ ranging from 0.11 to 0.18. In the validation cohort, the largest DMR was located in the *HTR2A* gene, within the transcription start site. This DMR contains 5 DMPs located within 211 base pairs with a Δ_meth_ ranging from − 0.10 to 0.17 (Table 3). On comparison between the two cohorts, three DMRs (located in *HTR2A*, *HDAC4* and *SLC17A9*) were replicated and had Δ_meth_ in a consistent direction (Table 2, 3 asterisk).

**Table 2:** DMRs in discovery cohort.

CpG	CHR	MAPINFO	Mean control	Mean MS	δετλα beta	P-value	Gene	CpGs in DMR	Feature	cgi
cg02267270	6	37,616,410	0.42	0.53	0.11	0.01	MDGA1	4	Body	Island
cg14926196	6	37,616,482	0.33	0.51	0.18	0.00	MDGA1	4	Body	Island
cg20053110	6	37,617,864	0.53	0.64	0.11	0.04	MDGA1	4	Body	Island
cg24796644	6	37,617,956	0.57	0.70	0.13	0.01	MDGA1	4	Body	Island
cg23299919	7	157,406,096	0.19	0.30	0.11	0.01	PTPRN2	3	Body	Island
cg06715136	7	158,046,025	0.73	0.83	0.11	0.02	PTPRN2	3	Body	Opensea
cg09756125	7	158,250,978	0.66	0.53	− 0.14	0.02	PTPRN2	3	Body	Shore
*cg18200810	13	47,472,200	0.60	0.48	− 0.11	0.02	HTR2A	3	TSS1500	Opensea
*cg24118521	13	47,472,330	0.74	0.58	− 0.16	0.02	HTR2A	3	TSS1500	Opensea
*cg23881368	13	47,472,343	0.68	0.55	− 0.13	0.01	HTR2A	3	TSS1500	Opensea
cg07075026	17	47,091,521	0.48	0.38	− 0.10	0.01	IGF2BP1	3	Body	Island
cg10950924	17	47,092,072	0.56	0.46	− 0.10	0.00	IGF2BP1	3	Body	Island
cg18128536	17	47,092,178	0.59	0.48	− 0.11	0.00	IGF2BP1	3	Body	Island
cg12099423	20	61,590,751	0.26	0.36	0.11	0.00	SLC17A9	3	Body	Shore
*cg17221813	20	61,590,823	0.38	0.51	0.13	0.00	SLC17A9	3	Body	Island
*cg19142181	20	61,591,066	0.37	0.54	0.17	0.00	SLC17A9	3	Body	Island
*cg07673080	2	240,241,154	0.89	0.76	− 0.12	0.00	HDAC4	2	Body	Opensea
*cg10639368	2	240,241,218	0.80	0.70	− 0.11	0.00	HDAC4	2	Body	Opensea
cg18850127	7	39,170,497	0.65	0.51	− 0.14	0.01	POU6F2	2	Body	Opensea
cg20302533	7	39,170,763	0.56	0.42	− 0.14	0.00	POU6F2	2	Body	Opensea
cg06238316	19	21,265,364	0.22	0.37	0.15	0.00	ZNF714	2	5′UTR	Island
cg09352518	19	21,265,421	0.24	0.44	0.20	0.00	ZNF714	2	5′UTR	Island
cg17099656	22	47,135,171	0.17	0.30	0.13	0.00	CERK	2	TSS1500	Opensea
cg16154810	22	47,135,258	0.35	0.55	0.20	0.00	CERK	2	TSS1500	Opensea

**Table 3:** DMRs in validation cohort.

CpG	CHR	MAPINFO	Mean control	Mean MS	deltaBeta	P.Value	gene	DMR	feature	cgi
cg06020661	13	47,472,138	0.75	0.65	− 0.10	0.01	HTR2A	5	TSS1500	Opensea
*cg18200810	13	47,472,200	0.60	0.47	− 0.14	0.03	HTR2A	5	TSS1500	Opensea
*cg24118521	13	47,472,330	0.74	0.57	− 0.17	0.03	HTR2A	5	TSS1500	Opensea
*cg23881368	13	47,472,343	0.69	0.54	− 0.15	0.02	HTR2A	5	TSS1500	Opensea
cg05506829	13	47,472,349	0.80	0.69	− 0.11	0.02	HTR2A	5	TSS1500	Opensea
cg26371957	12	739,280	0.67	0.80	0.13	0.03	NINJ2	4	Body	Opensea
cg14911689	12	739,980	0.28	0.50	0.22	0.02	NINJ2	4	Body	Opensea
cg26654770	12	740,100	0.28	0.55	0.27	0.02	NINJ2	4	Body	Opensea
cg01201512	12	740,338	0.37	0.60	0.23	0.04	NINJ2	4	Body	Opensea
*cg07673080	2	2.4E+08	0.88	0.76	− 0.11	0.01	HDAC4	2	Body	Opensea
*cg10639368	2	2.4E+08	0.80	0.69	− 0.10	0.02	HDAC4	2	Body	Opensea
cg11938672	6	1.7E+08	0.71	0.59	− 0.13	0.01	WDR27	2	Body	Opensea
cg18322025	6	1.7E+08	0.63	0.47	− 0.16	0.01	WDR27	2	Body	Shelf
cg18020065	13	1.15E+08	0.34	0.47	0.13	0.01	RASA3	2	Body	Opensea
cg16820615	13	1.15E+08	0.73	0.62	− 0.11	0.00	RASA3	2	Body	Opensea
*cg17221813	20	61,590,823	0.39	0.50	0.11	0.03	SLC17A9	2	Body	Island
*cg19142181	20	61,591,066	0.39	0.54	0.15	0.05	SLC17A9	2	Body	Island

**Figure 1:**
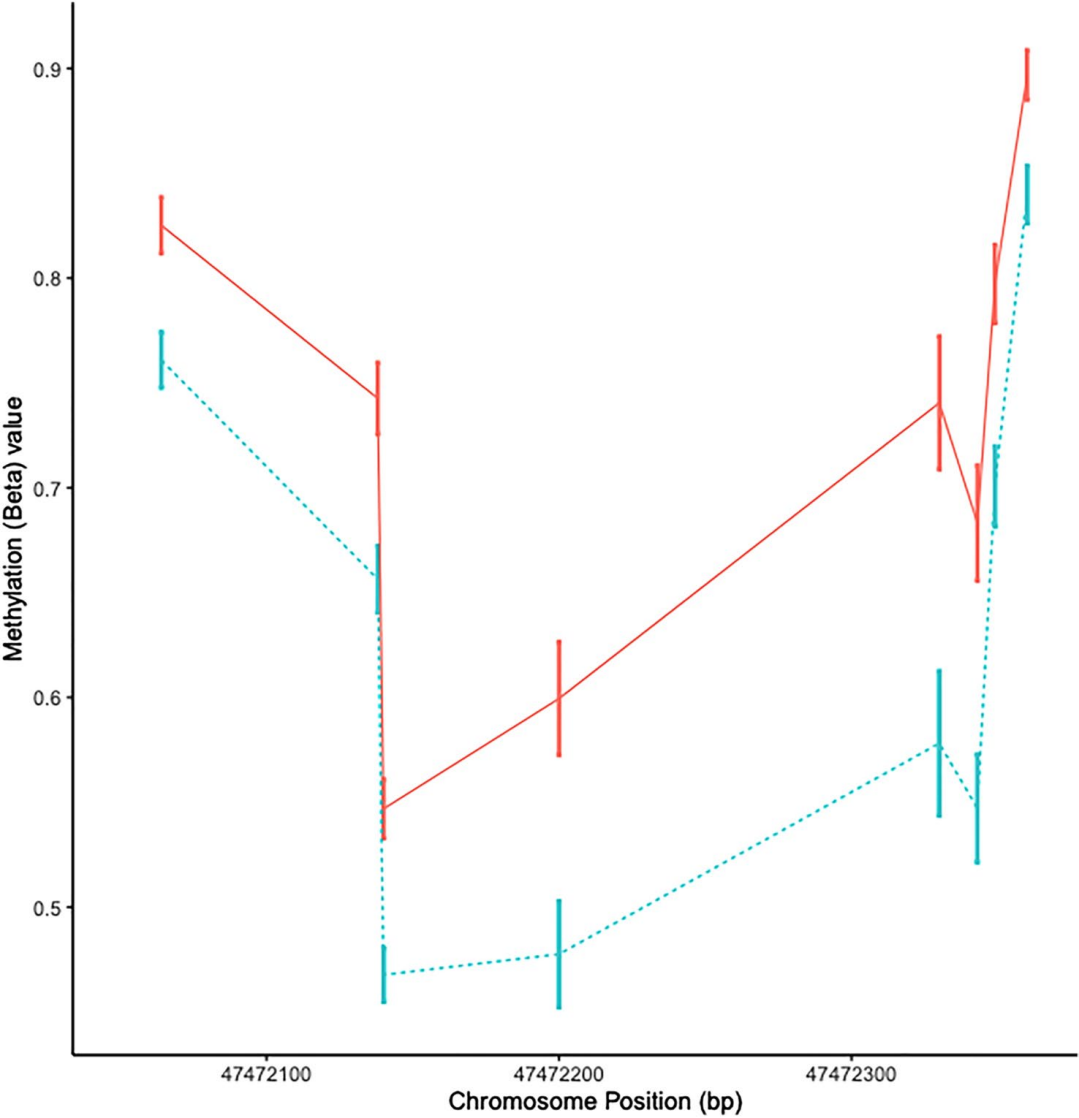
HTR2A DMR plot. The mean beta value at the eight *HTR2A* CpG sites for controls (solid red line) and SPMS patients (dotted blue line) located within the transcription start site. Error bars show standard error of the mean.

DNA methylation is most likely to exert an effect on expression within the transcriptional start site (TSS). The only DMR identified both in the TSS and in both cohorts was the DMR at *HTR2A*. Therefore, to gain a more comprehensive profile of the *HTR2A* DMR we joined the two cohorts together and re-analysed all CpGs in *HTR2A* for differential methylation between cases and controls. In this analysis there were eight CpGs exhibiting > 5% hypomethylation in the cases compared to controls (P < 0.005) (Table S3). These 8 DMPs are located within a 283 base pair region in the TSS1500 of the gene (Fig. 1). The strongest association was observed for cg09798090 (Δ_meth_ = − 0.08, P = 0.00009). There was a strong positive correlation among all 8 CpGs in this cohort supporting this as a single DMR (r $\ge$ 0.85, P < 0.0001) (Suppl. Fig. S3). The probe cg09798090 was selected as the index (or tagging) CpG for subsequent analyses.

### The *HTR2A* DMR is contained within the bounds of a haplotype block

**Figure 2:**
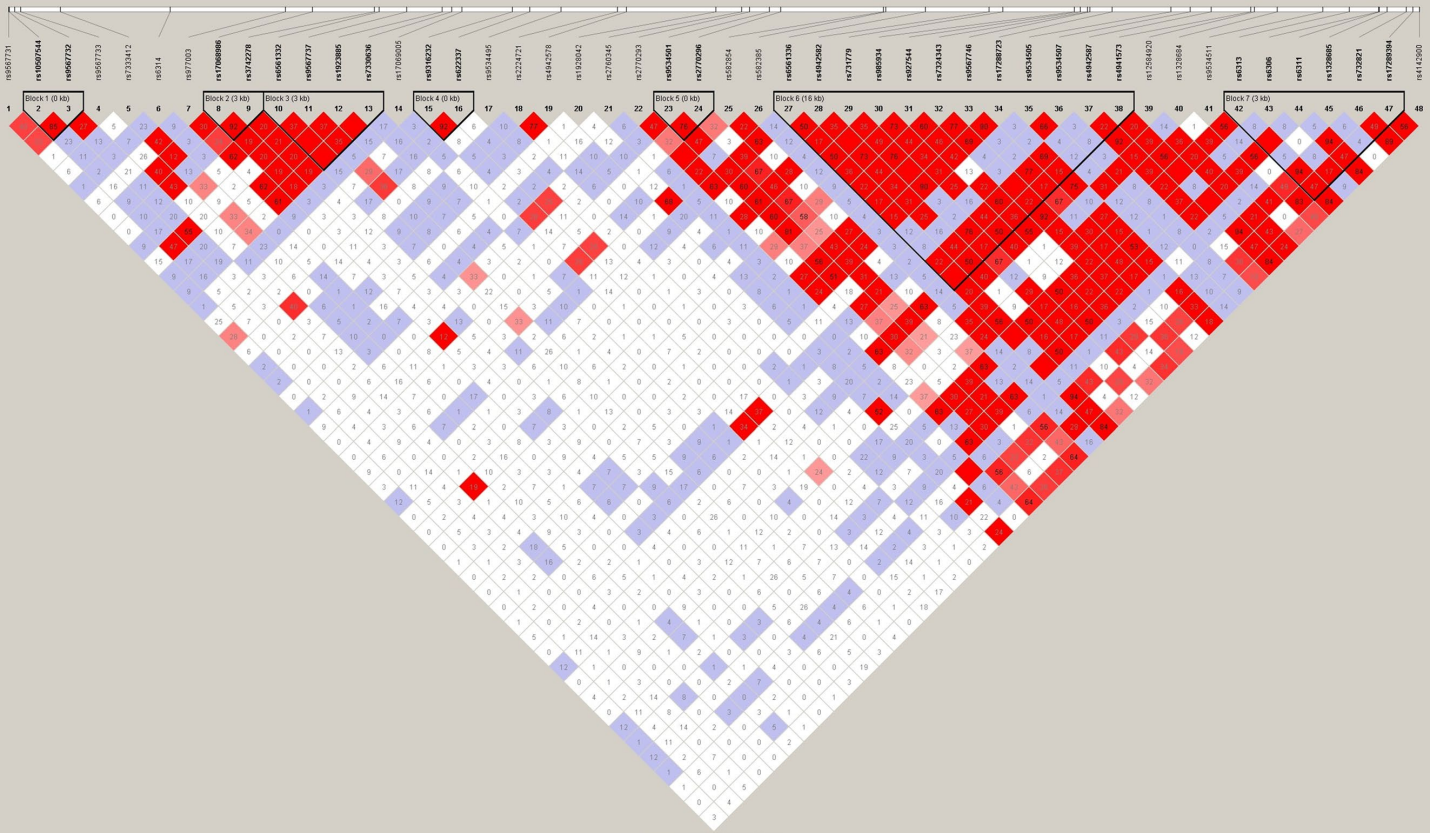
Haplotype block at HTR2A. Haploview chart showing the 7 haplotype blocks at *HTR2A*. Blocks are indicated by a bold black outline. SNPs contained within the block are in bold and the length of the block is indicated (in base pairs) in parentheses. This plot provides information on both the r^2^ and D′ values for better clarification of any linkage disequilibrium. The number within each diamond represents the r^2^ value between each diagonally corresponding SNP pairs and the colour of each diamond represents the strength/value of D′ (white to bright red/0 < 100).

A previous study, focused on schizophrenia, demonstrated that both gene expression and methylation at *HTR2A* are likely under genetic control^27^. To investigate if this is the case in our cohort, we performed a haplotype block analysis using Haploview. The HumanOmniExpress-24 arrays contain 50 SNPs for the *HTR2A* gene. We identified 7 haplotype blocks in this cohort (Fig. 2). The DMR is located within block 7 between rs1328685 and rs732821 and is tagged by rs6313, previously linked to a number of other inflammatory and neurological diseases^28,29^. Even in this modestly sized sample there was evidence of association between rs6313 and progressive MS. In particular, patients were more likely to carry the risk allele (C) compared to controls (OR 5.3, P = 0.028).

To determine if this DMR also resulted in expression level changes, we examined the *HTR2A* probe of the HT12 arrays between HC and MS patients. The HT12 arrays only have one probe for *HTR2A*, which is lowly expressed in both HC and MS patients (according to manufacturer’s manifest/product information). There were no identified changes between HC and progressive MS patients.

### Methylation quantitative trait loci (mQTL) analysis

**Figure 3:**
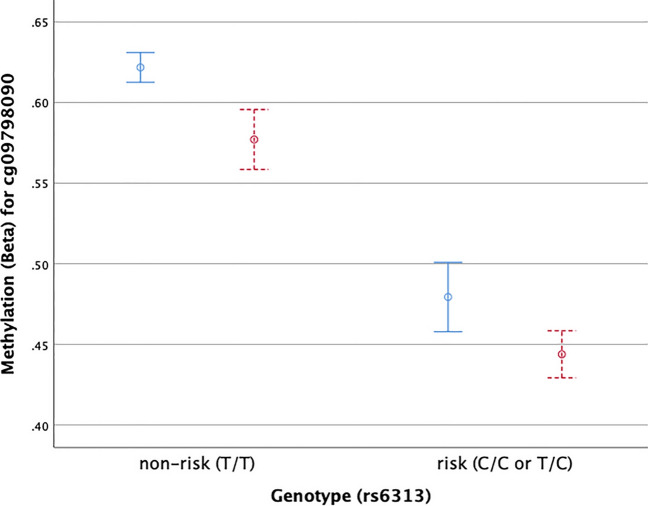
HTR2A mQTL. Mean delta beta values at cp09798090 for SNP rs6313 in either the non-risk allele (T/T) or risk allele (C/C or T/C). Healthy controls (HC) are shown in blue, SPMS are shown in read. Error bars are standard error of the mean.

The effect of genetic variants on disease phenotypes can be mediated by DNA methylation via mQTLs. To assess this in our cohort we compared the average methylation of the index CpG (cg09798090) across the genotype groups for the index SNP (rs6313). Figure 3 shows a highly significant differences in methylation levels between risk allele carriers and non-carriers (P < 0.0001). Figure 3 also indicates that the hypomethylation in progressive patients may occur independently of genotype. To explore this, we performed a logistic regression with phenotype as the outcome and SNP (rs6313) and CpG (cg09798090) as joint predictors. Results of this model showed that the DMR (as represented by cg09798090) remained significantly associated with MS after accounting for rs6313 genotype (P = 0.04).

### *HTR2A* is not differentially methylated in RRMS samples

In order to identify if this mQTL is specific to progressive MS, we returned to our original RRMS cohort^6^ and reanalysed the data using the same methods as above. There was no evidence for differential methylation in *HTR2A.* Similarly, there were no statistically significant differences between the risk allele frequency in the HC and the RRMS populations (Suppl. Table S4).

## Discussion

In this study we report the results of an EWAS of methylation levels in the CD4^+^ T cells of female progressive MS patients compared to age and gender-matched healthy controls. We identified a DMR upstream of *HTR2A* in the TSS, which is located within the bounds of a haplotype block. Neither the DMR, nor the haplotype block are associated with RRMS and therefore appear to be progressive MS specific.

*HTR2A* codes for the serotonin 2A receptor. Serotonin is a neurotransmitter that acts in the gastrointestinal system, the blood and the brain. SNPs located within *HTR2A* have been associated with numerous neuropsychiatric disorders (including schizophrenia and obsessive–compulsive disorder) as well as autoimmune disease (rheumatoid arthritis)^29,30^. Although whether these SNPs contribute to disease has been recently challenged^31^. The most well studied SNP, rs6311 is known to be in near complete linkage disequilibrium with rs6313 and rs732821^32^. It is therefore not surprising that two of the three SNPs we identified in this study were rs6311 and 6313.

Expression of *HTR2A* is known to be controlled in the brain by functional polymorphisms, particularly rs6313 (102T/C allele) and rs6311 (-14378A/G allele). However, there is also evidence that low-level expression occurs in the peripheral lymphocytes^27,33^; although, there seems to be discrepancy over whether this expression is mono- or biallelic. In this study, we assessed the expression of *HTR2A* in our discovery cohort and found no differences between healthy controls and MS patients. This could be due to low expression in CD4^+^ T cells. Previous studies assessed a mixed population of PBMCs, therefore, it is possible that expression was driven primarily by a cell population other than CD4^+^ T cells^27,33^. Alternately, the HT12 arrays only have a single probe for *HTR2A*. Studies, have suggested that there are alternative splice variants for *HTR2A,* which are not detected by the single probe available on the HT12 array. Future studies using next-generation sequencing technologies will be beneficial to answer these questions. Additional studies, which also evaluate other cell types will also be beneficial to determine if the methylation changes in CD4^+^ T cells are reflective of changes in other cells. This would be particularly interesting in CNS resident cells.

The two genetic variants (rs6311 and rs6313) have also been shown to affect methylation status of *HTR2A* in other conditions such as schizophrenia and autism^34^. The minor A allele of rs6311 changes the CpG site to a CpA site, thus reducing the methylation in the promoter region^35,36^. This is also the case for the HLA-DRB1*1501 allele in MS and for the rs6311 SNP in other diseases. Our logistic regression analysis suggests that the DMR at *HTR2A* exists regardless of rs6311 genotype. Although significance is marginal, these results suggest that methylation at the *HTR2A* gene are not completely driven by genotype and may be influenced by other factors in MS. Furthermore, comparison to our previous data sets, suggests that the DMR is progressive MS specific and supports the notion that *HTR2A* genotype is not the sole regulator of methylation at this locus in MS.

The link between MS and *HTR2A* is unclear. However, Malinova et al. reviewed the literature on serotonin and MS and point to a strong link between serotonin (5-HT) and gut microbiome^37^. Platelets normally take up 5HT in the gut, transport it to the peripheral blood, where it is secreted and transforms CD4^+^ T cells into Th1 class cells^37^. In MS patients, the secretion fails to happen, thereby linking 5HT to disease activation.

Although we chose to focus on the *HTR2A* gene, due to its location within the TSS, we did also identify two other DMRs as part of this study in *HDAC4* and *SLC17A9*. Histone Deacetylase 4 is a class IIa histone deacetylase which is ubiquitously expressed throughout the body^38^. HDACs have a well-established roll in cell growth and differentiation. HDAC4 may play a role in neuronal plasticity, and there is some evidence that HDAC inhibitors lessen the neuropathy of mice with experimental models of MS (EAE)^39^. But a direct link between HDAC4 and MS in humans has not been described.

Solute Carrier Family 17 member 9A (SLC179A) codes for a vesicular nucleotide transporter that plays a role in storage and secretion of adenosine triphosphate (ATP). One study investigating normal appearing white matter (NAWM) of MS patients’ brain tissue identified that SLC17A9 was hypomethylated in patients compared to healthy controls. This corresponded with an upregulation of the RNA transcript^40^. The second study, investigated the role of hypoxia in the pathophysiology of periplaque demyelinated lesions (PDL) in MS by comparing the PDL to NAWM^41^. They found that SLC179A was a hypoxia-inducible factor (HIF-1a) target gene that was upregulated in PDLs, suggesting that it may play a role in hypoxia induced demyelination^41^.

One limitation of the study is that the patients had a varied and complex treatment history. We cannot exclude that treatment is affecting our results and, given the small sample numbers, we are also not able to perform sub-cohort analysis. Additionally, two different arrays were used for this study, the 450K and EPIC arrays. The EPIC arrays contain nearly twice as many probes as the original 450K arrays, so we are unable to validate the findings from the extra probes. However, future larger studies with the EPIC arrays will help to further validate our findings.

Finally, there are currently no biomarkers of progression, and progressive MS is diagnosed retrospectively^16^. The absence of the *HTR2A* DMR in our previous cohort of RRMS patients suggests that this DMR may be specific to progressive disease, making it a potential biomarker of progression.

## Supplementary Information

Supplementary Information.

## Data Availability

The dataset supporting the conclusions of this article is included in the supplemental files. For access to raw datasets contact Rodney Lea.

## References

[1] Segal BM (2014). Stage-specific immune dysregulation in multiple sclerosis. J. Interferon Cytokine Res..

[2] Broux B, Stinissen P, Hellings N (2013). Which immune cells matter? The immunopathogenesis of multiple sclerosis. Crit. Rev. Immunol..

[3] Palmer AJ, Colman S, O'Leary B, Taylor BV, Simmons RD (2013). The economic impact of multiple sclerosis in Australia in 2010. Mult. Scler..

[4] Zhu H (2013). A genome-wide methylation study of severe vitamin D deficiency in African American adolescents. J. Pediatr..

[5] Wan ES (2012). Cigarette smoking behaviors and time since quitting are associated with differential DNA methylation across the human genome. Hum. Mol. Genet..

[6] Maltby VE (2015). Genome-wide DNA methylation profiling of CD8+ T cells shows a distinct epigenetic signature to CD4+ T cells in multiple sclerosis patients. Clin. Epigenet..

[7] Maltby VE (2018). A hypermethylated region in the promoter of the lymphotoxin-alpha gene in CD19+ B cells is associated with multiple sclerosis. Mult. Scler. J..

[8] Maltby VE (2017). Differential methylation at MHC in CD4+ T cells is associated with multiple sclerosis independently of HLA-DRB1. Clin. Epigenet..

[9] Baranzini SE (2010). Genome, epigenome and RNA sequences of monozygotic twins discordant for multiple sclerosis. Nature.

[10] Bos SD (2015). Genome-wide DNA methylation profiles indicate CD8+ T cell hypermethylation in multiple sclerosis. PLoS ONE.

[11] Graves M (2013). Methylation differences at the HLA-DRB1 locus in CD4+ T-Cells are associated with multiple sclerosis. Mult. Scler..

[12] Ruhrmann S (2018). Hypermethylation of MIR21 in CD4+ T cells from patients with relapsing-remitting multiple sclerosis associates with lower miRNA-21 levels and concomitant up-regulation of its target genes. Mult. Scler..

[13] Kular L (2018). DNA methylation as a mediator of HLA-DRB1*15:01 and a protective variant in multiple sclerosis. Nat. Commun..

[14] Tintore M, Vidal-Jordana A, Sastre-Garriga J (2019). Treatment of multiple sclerosis—success from bench to bedside. Nat. Rev. Neurol..

[15] Thompson AJ (2017). Diagnosis of multiple sclerosis: 2017 revisions of the McDonald criteria. Lancet Neurol..

[16] Lorscheider J (2016). Defining secondary progressive multiple sclerosis. Brain.

[17] Tian Y (2017). ChAMP: updated methylation analysis pipeline for Illumina BeadChips. Bioinformatics.

[18] Aryee MJ (2014). Minfi: a flexible and comprehensive Bioconductor package for the analysis of Infinium DNA methylation microarrays. Bioinformatics.

[19] Pidsley R (2013). A data-driven approach to preprocessing Illumina 450K methylation array data. BMC Genomics.

[20] RStudio Team (2018). RStudio: Intergrated Development for R.

[21] R*: A Language and Environment for Statistical Computing* (R Foundation for Statistical Computing, Vienna, Austria, 2018).

[22] Chen YA (2013). Discovery of cross-reactive probes and polymorphic CpGs in the Illumina Infinium HumanMethylation450 microarray. Epigenetics.

[23] Barrett JC, Fry B, Maller J, Daly MJ (2005). Haploview: analysis and visualization of LD and haplotype maps. Bioinformatics.

[24] Ritchie ME (2015). limma powers differential expression analyses for RNA-sequencing and microarray studies. Nucleic Acids Res..

[25] Smith ML, Baggerly KA, Bengtsson H, Ritchie ME, Hansen KD (2013). illuminaio: an open source IDAT parsing tool for Illumina microarrays. F1000Res.

[26] Kebschull M, Fittler MJ, Demmer RT, Papapanou PN (2017). Differential expression and functional analysis of high-throughput -omics data using open source tools. Methods Mol. Biol..

[27] Fukuda Y (2006). Monoallelic and unequal allelic expression of the HTR2A gene in human brain and peripheral lymphocytes. Biol. Psychiatry.

[28] Petit AC (2014). Converging translational evidence for the involvement of the serotonin 2A receptor gene in major depressive disorder. Prog. Neuropsychopharmacol. Biol. Psychiatry.

[29] Williams J (1996). Association between schizophrenia and T102C polymorphism of the 5-hydroxytryptamine type 2a-receptor gene. European Multicentre Association Study of Schizophrenia Group. Lancet.

[30] Mattina GF, Samaan Z, Hall GB, Steiner M (2020). The association of HTR2A polymorphisms with obsessive-compulsive disorder and its subtypes: a meta-analysis. J. Affect. Disord..

[31] Spies M (2020). Common HTR2A variants and 5-HTTLPR are not associated with human in vivo serotonin 2A receptor levels. Hum. Brain Mapp..

[32] Spurlock G (1998). A family based association study of T102C polymorphism in 5HT2A and schizophrenia plus identification of new polymorphisms in the promoter. Mol. Psychiatry.

[33] Guhathakurta S (2009). Analysis of serotonin receptor 2A gene (HTR2A): association study with autism spectrum disorder in the Indian population and investigation of the gene expression in peripheral blood leukocytes. Neurochem. Int..

[34] Hranilovic D, Blazevic S, Stefulj J, Zill P (2016). DNA methylation analysis of HTR2A regulatory region in leukocytes of autistic subjects. Autism Res..

[35] Parsons MJ, D'Souza UM, Arranz MJ, Kerwin RW, Makoff AJ (2004). The -1438A/G polymorphism in the 5-hydroxytryptamine type 2A receptor gene affects promoter activity. Biol. Psychiatry.

[36] Turecki G (1999). Prediction of level of serotonin 2A receptor binding by serotonin receptor 2A genetic variation in postmortem brain samples from subjects who did or did not commit suicide. Am. J. Psychiatry.

[37] Malinova TS, Dijkstra CD, de Vries HE (2018). Serotonin: a mediator of the gut-brain axis in multiple sclerosis. Mult. Scler..

[38] Fischer A, Sananbenesi F, Mungenast A, Tsai LH (2010). Targeting the correct HDAC(s) to treat cognitive disorders. Trends Pharmacol. Sci..

[39] Camelo S (2005). Transcriptional therapy with the histone deacetylase inhibitor trichostatin A ameliorates experimental autoimmune encephalomyelitis. J. Neuroimmunol..

[40] Huynh JL (2014). Epigenome-wide differences in pathology-free regions of multiple sclerosis-affected brains. Nat. Neurosci..

[41] Satoh J, Asahina N, Kitano S, Kino Y (2015). Bioinformatics data mining approach indicates the expression of chromatin immunoprecipitation followed by deep sequencing (ChIP-Seq)-based hypoxia-inducible factor-1α target genes in periplaque lesions of multiple sclerosis. Clin. Exp. Neuroimmunol..

