# Supplementary material for: Epigenetic differences at the HTR2A locus in progressive multiple sclerosis patients
Source: Sci Rep. 2020 Dec 17;10:22217. doi: 10.1038/s41598-020-78809-x (PMC7747721; doi:10.1038/s41598-020-78809-x)
Supplement: Supplementary file 1 — Supplementary Information. [file 41598_2020_78809_MOESM1_ESM.pdf]

Epigenetic differences at the *HTR2A* locus in progressive multiple sclerosis patients

SUPPLEMENTARY FIGURES/TABLES

Vicki E Maltby[1, 2]#, Rodney A Lea[2, 3]#, Sean Burnard[2,4], Alexandre Xavier[2,4], Thao Van Cao[3] Nicole White[3], Daniel Kennedy[3], Kira Groen[1, 2], Katherine A. Sanders[2,5], Rebecca Seeto[1,6], Samara Bray[1,2], Melissa Gresle[7,8,10], Louise Laverick[7,8], Helmut Butzkueven[9,10], Rodney J Scott[4,11,12], Jeannette Lechner-Scott[1,2,13]*

1. School of Medicine and Public Health, University of Newcastle, Callaghan NSW 2308, Australia
2. Centre for Brain and Mental Health, Hunter Medical Research Institute, New Lambton Heights NSW 2305, Australia
3. Institute of Health and Biomedical Innovations, Genomics Research Centre, Queensland University of Technology, Kelvin Grove QLD 4059, Australia
4. School of Biomedical Sciences and Pharmacy, University of Newcastle, Callaghan NSW 2308, Australia
5. Centre for Anatomical and Human Sciences, Hull York Medical School, Hull UK.
6. School of Environmental and Life Sciences, University of Newcastle, Callaghan, NSW, 2308, Australia
7. Department of Medicine, University of Melbourne, Melbourne VIC Australia.
8. Royal Melbourne Hospital, Melbourne VIC, Australia
9. Alfred Hospital, Melbourne VIC, Australia
10. MS and Neuroimmunology Unit, Central Clinical School, Monash University, Melbourne VIC, Australia
11. Division of Molecular Genetics, Pathology North, John Hunter Hospital, New Lambton Heights NSW 2305, Australia
12. Centre for Cancer Research, Hunter Medical Research Institute, New Lambton Heights NSW 2305, Australia
13. Department of Neurology, John Hunter Hospital, New Lambton Heights NSW 2305, Australia

Supplementary Figures 1a

QC assessments on raw data for both cohorts. Beta density plots for discovery (panel A) and validation (panel B) cohorts with controls (green) and MS patients (orange). Multiple Dimensional Scaling (MDS) plots for discovery (panel C) and validation (panel D) cohorts) with controls (green) and MS patients (orange). Sex check plots for discovery (panel E) and validation (panel F) cohorts for female (pink) and male (blue).

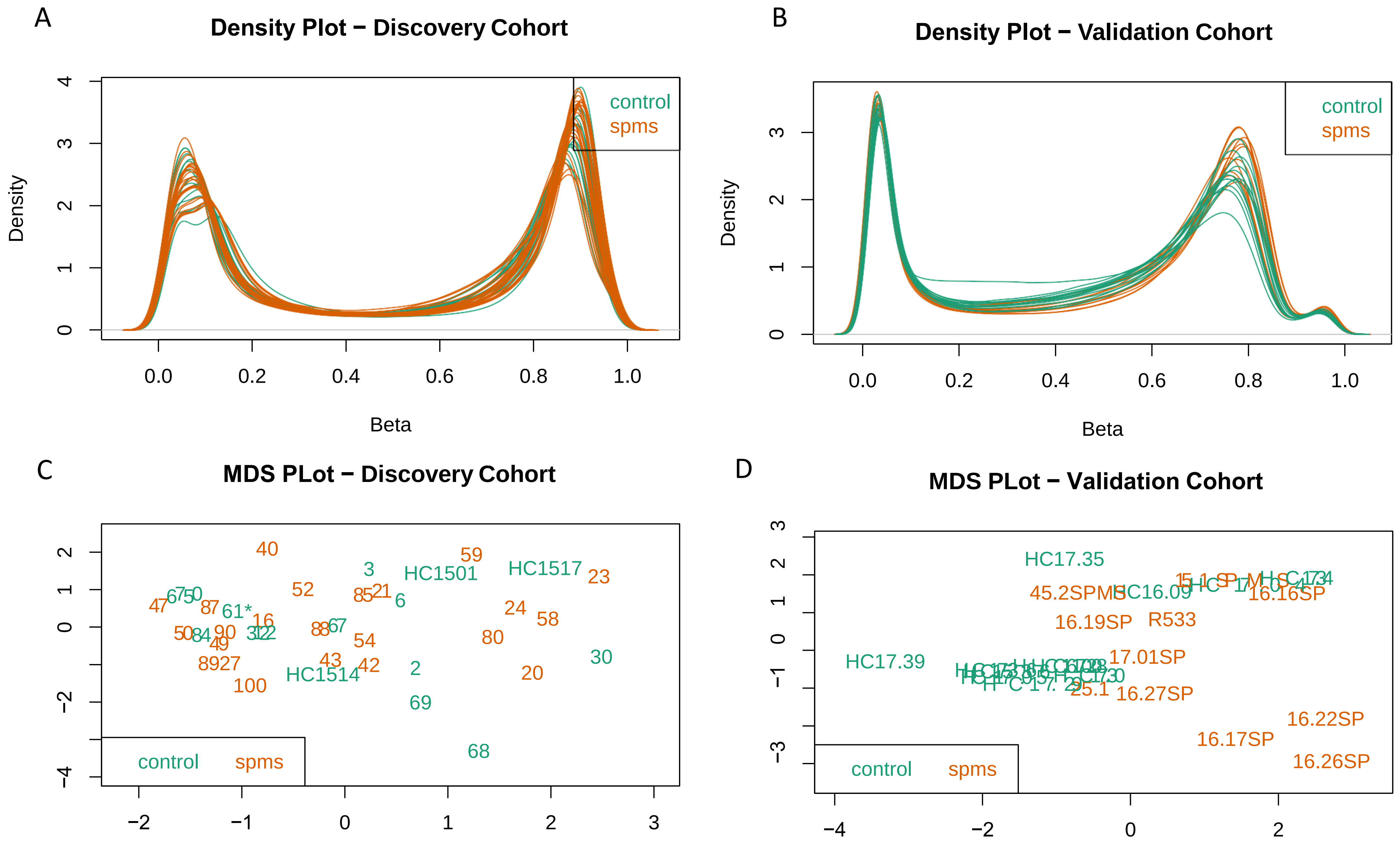

A
Density Plot − Discovery Cohort
Density
Beta
control
spms
B
Density Plot − Validation Cohort
Density
Beta
control
spms
C
MDS PLot − Discovery Cohort
control
spms
D
MDS PLot − Validation Cohort
control
spms
HC17.35
HC17.39
45.2SPMS
HC16.09
16.16SP
16.19SP
R533
17.01SP
16.27SP
16.22SP
16.17SP
16.26SP
HC1501
HC1517
HC1514

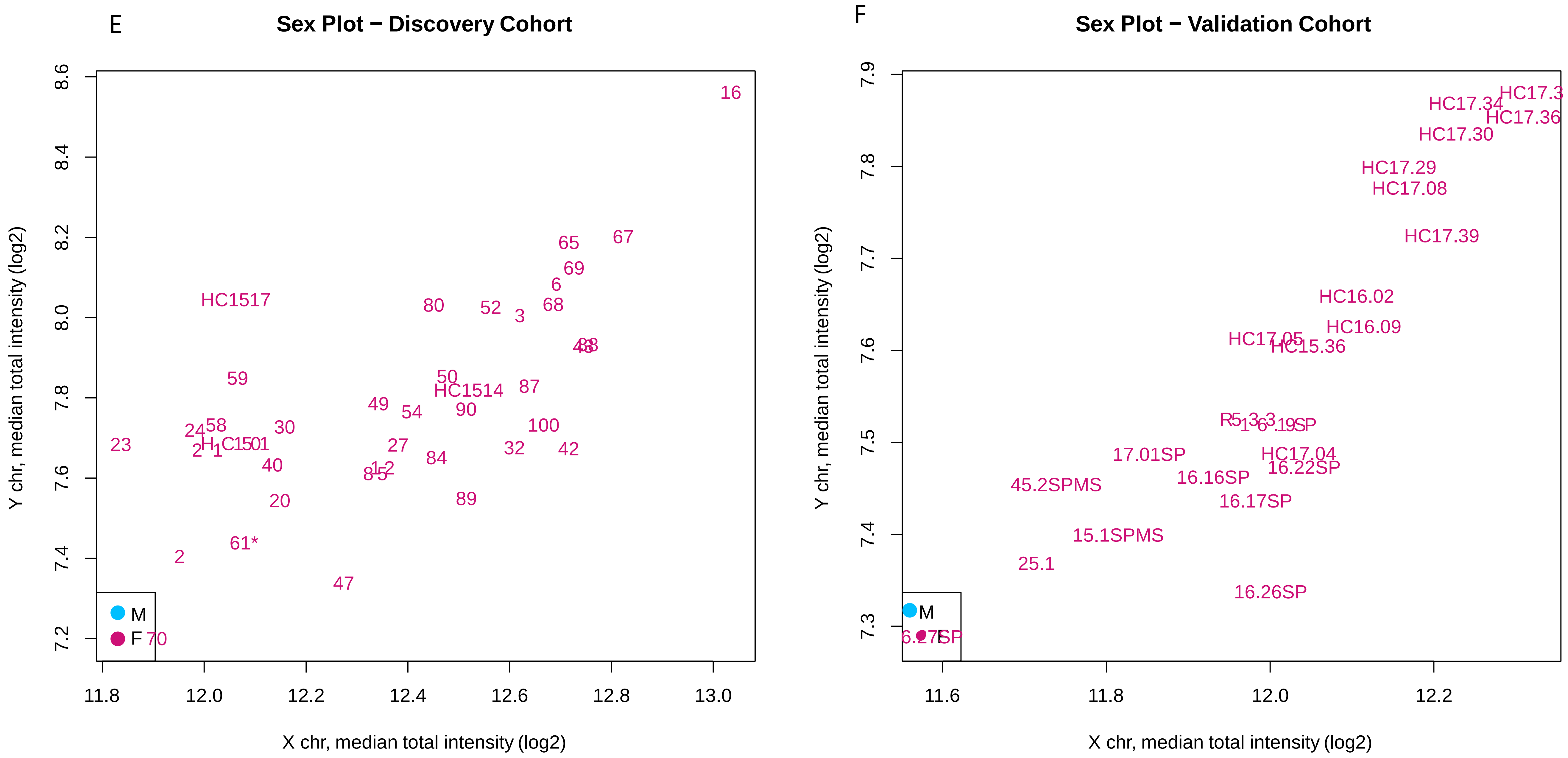
E
Sex Plot − Discovery Cohort
Y chr, median total intensity (log2)
X chr, median total intensity (log2)
M
F
F
Sex Plot − Validation Cohort
Y chr, median total intensity (log2)
X chr, median total intensity (log2)
M
F

Supplementary Files 1b

QC assessments on normalized data for both cohorts. Beta density plots for discovery (panel A) and validation (panel B) cohorts with controls (green) and MS patients (orange). Multiple Dimensional Scaling (MDS) plots for discovery (panel C) and validation (panel D) cohorts) with controls (green) and MS patients (orange).

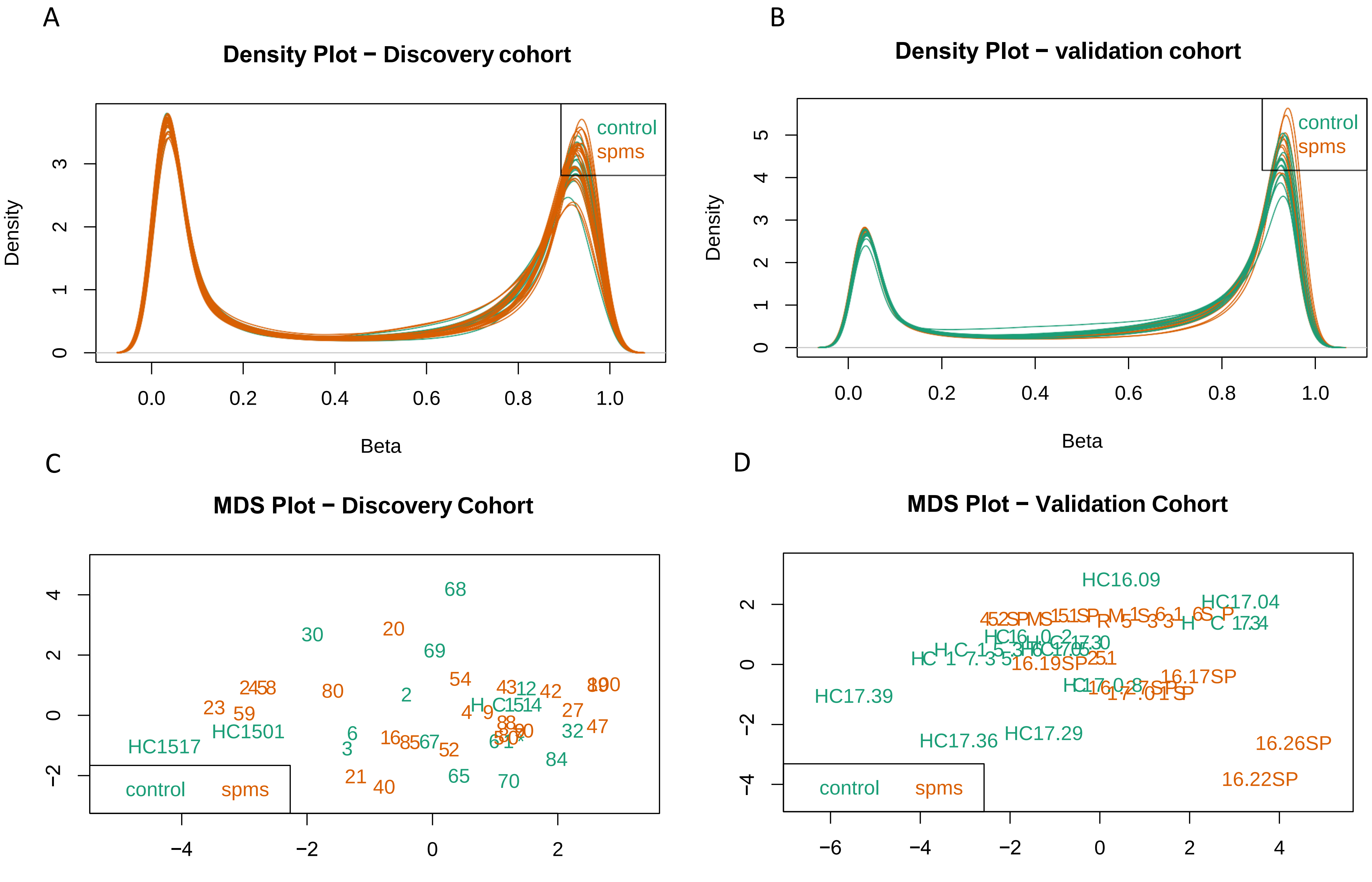
A
Density Plot − Discovery cohort
Density
Beta
control
spms
B
Density Plot − validation cohort
Density
Beta
control
spms
C
MDS Plot − Discovery Cohort
control
spms
D
MDS Plot − Validation Cohort
control
spms

Supplementary Files 2

Batch effect analysis for both cohorts combined left panel is before correction, right panel shows batch effects post corrections

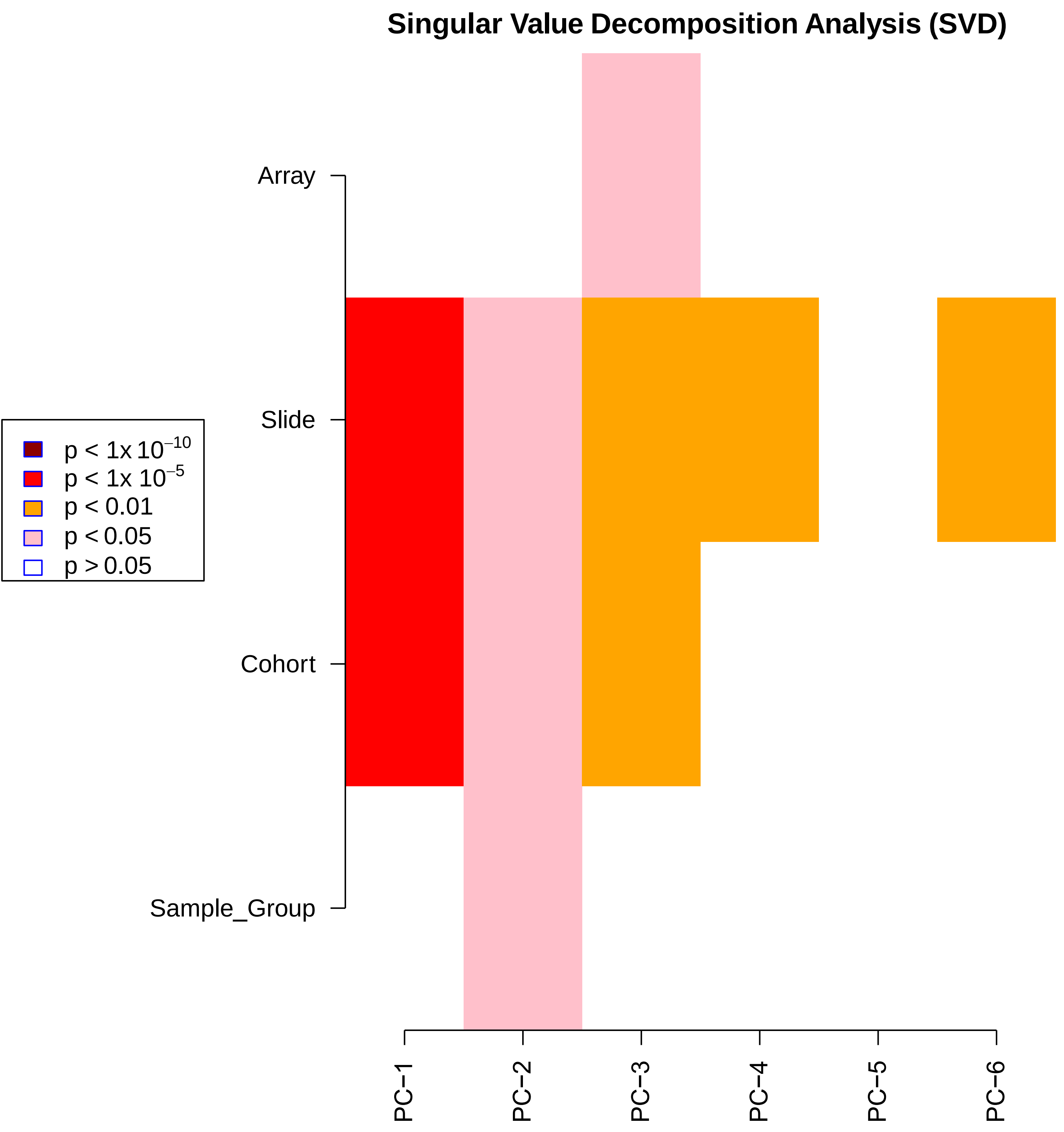

Singular Value Decomposition Analysis (SVD)
Array
Slide
Cohort
Sample_Group
PC−1
PC−2
PC−3
PC−4
PC−5
PC−6
p < 1x 10−10
p < 1x 10−5
p < 0.01
p < 0.05
p > 0.05

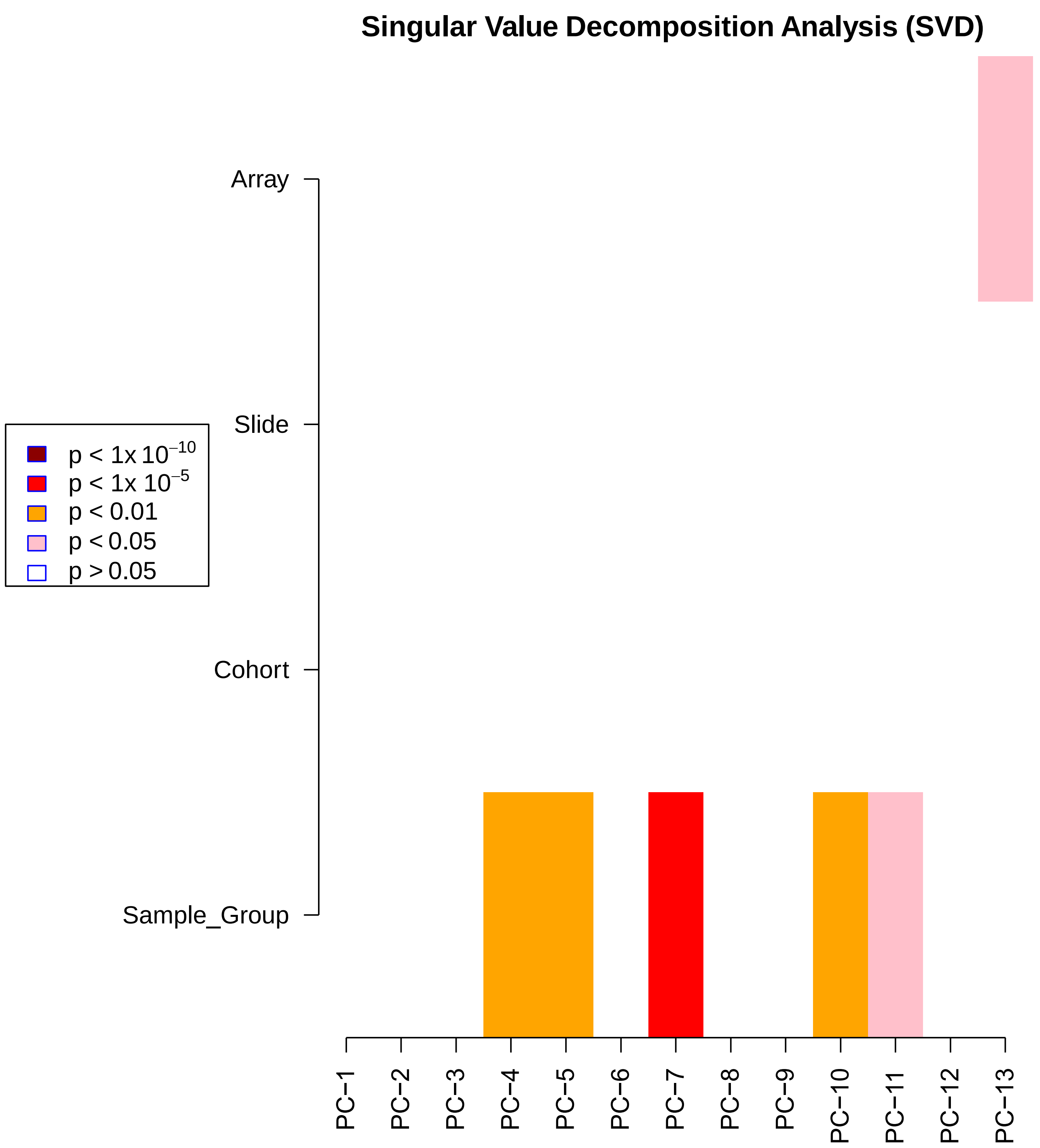

Singular Value Decomposition Analysis (SVD)
Array
Slide
Cohort
Sample_Group
PC−1
PC−2
PC−3
PC−4
PC−5
PC−6
PC−7
PC−8
PC−9
PC−10
PC−11
PC−12
PC−13
p < 1x 10−10
p < 1x 10−5
p < 0.01
p < 0.05
p > 0.05

Supplementary Table 3 HTR2A CpG Correlation matrix

Correlations

		cg01620540	cg06020661	cg09798090	cg18200810	cg24118521	cg23881368	cg05506829	cg07075299
cg01620540	Pearson Correlation	1	.895**	.890**	.887**	.883**	.866**	.859**	.892**
	Sig. (2-tailed)		.000	.000	.000	.000	.000	.000	.000
	N	35	35	35	35	35	35	35	35
cg06020661	Pearson Correlation	.895**	1	.988**	.976**	.978**	.980**	.972**	.926**
	Sig. (2-tailed)	.000		.000	.000	.000	.000	.000	.000
	N	35	35	35	35	35	35	35	35
cg09798090	Pearson Correlation	.890**	.988**	1	.980**	.975**	.977**	.967**	.915**
	Sig. (2-tailed)	.000	.000		.000	.000	.000	.000	.000
	N	35	35	35	35	35	35	35	35
cg18200810	Pearson Correlation	.887**	.976**	.980**	1	.987**	.985**	.976**	.919**
	Sig. (2-tailed)	.000	.000	.000		.000	.000	.000	.000
	N	35	35	35	35	35	35	35	35
cg24118521	Pearson Correlation	.883**	.978**	.975**	.987**	1	.988**	.976**	.933**
	Sig. (2-tailed)	.000	.000	.000	.000		.000	.000	.000
	N	35	35	35	35	35	35	35	35
cg23881368	Pearson Correlation	.866**	.980**	.977**	.985**	.988**	1	.979**	.923**
	Sig. (2-tailed)	.000	.000	.000	.000	.000		.000	.000
	N	35	35	35	35	35	35	35	35
cg05506829	Pearson Correlation	.859**	.972**	.967**	.976**	.976**	.979**	1	.909**
	Sig. (2-tailed)	.000	.000	.000	.000	.000	.000		.000
	N	35	35	35	35	35	35	35	35
cg07075299	Pearson Correlation	.892**	.926**	.915**	.919**	.933**	.923**	.909**	1
	Sig. (2-tailed)	.000	.000	.000	.000	.000	.000	.000	
	N	35	35	35	35	35	35	35	35

**. Correlation is significant at the 0.01 level (2-tailed).

Supplementary Table 4

			SPMS				RRMS			
Present_in	SNP	BP	F_A.CD4	F_U.CD4	P.CD4	OR.CD4	F_A.CD4	F_U.CD4	P.CD4	OR.CD4
CD4	rs1328684	47466230	0.2273	0.5385	0.0104	0.2521	0.375	0.3235	0.6486	1.255
CD4	rs9534495	47429228	0.5	0.1923	0.01226	4.2	0.4583	0.4412	1	1.072
Both	rs1928042	47437216	0.1136	0.3846	0.0141	0.2051	0.2292	0.25	1	0.8919
CD4	rs732821	47472879	0.5455	0.2692	0.02826	3.257	0.4167	0.4118	1	1.02
CD4	rs6561336	47448060	0.5227	0.2692	0.04789	2.973	0.3958	0.3824	1	1.058
Both	rs4941573	47464857	0.5227	0.2692	0.04789	2.973	0.3958	0.3824	1	1.058
CD4	rs6313	47469940	0.5227	0.2692	0.04789	2.973	0.3958	0.3824	1	1.058
CD4	rs6311	47471478	0.5227	0.2692	0.04789	2.973	0.3958	0.3824	1	1.058
Both	rs4142900	47473550	0.5682	0.3077	0.04807	2.961	0.5	0.4412	0.6574	1.267
CD4	rs2760345	47438574	0.02273	0.1538	0.05979	0.1279	0.1042	0.1176	1	0.8721
CD4	rs1328685	47471890	0.02273	0.1538	0.05979	0.1279	0.125	0.1176	1	1.071
CD4	rs6314	47409034	0.1364	0	0.07767	NA	0.02083	0.05882	0.5669	0.3404
Both	rs977003	47415001	0.5682	0.3462	0.08758	2.485	0.4167	0.4412	1	0.9048
